# Does Habitual Physical Activity Increase the Sensitivity of the Appetite Control System? A Systematic Review

**DOI:** 10.1007/s40279-016-0518-9

**Published:** 2016-03-22

**Authors:** Kristine Beaulieu, Mark Hopkins, John Blundell, Graham Finlayson

**Affiliations:** 1 School of Psychology, University of Leeds, Leeds, LS2 9JT UK; 2 Academy of Sport and Physical Activity, Sheffield Hallam University, Sheffield, UK

## Abstract

**Background:**

It has been proposed that habitual physical activity improves appetite control; however, the evidence has never been systematically reviewed.

**Objective:**

To examine whether appetite control (e.g. subjective appetite, appetite-related peptides, food intake) differs according to levels of physical activity.

**Data Sources:**

Medline, Embase and SPORTDiscus were searched for articles published between 1996 and 2015, using keywords pertaining to physical activity, appetite, food intake and appetite-related peptides.

**Study Selection:**

Articles were included if they involved healthy non-smoking adults (aged 18–64 years) participating in cross-sectional studies examining appetite control in active and inactive individuals; or before and after exercise training in previously inactive individuals.

**Study Appraisal and Synthesis:**

Of 77 full-text articles assessed, 28 studies (14 cross-sectional; 14 exercise training) met the inclusion criteria.

**Results:**

Appetite sensations and absolute energy intake did not differ consistently across studies. Active individuals had a greater ability to compensate for high-energy preloads through reductions in energy intake, in comparison with inactive controls. When physical activity level was graded across cross-sectional studies (low, medium, high, very high), a significant curvilinear effect on energy intake (z-scores) was observed.

**Limitations:**

Methodological issues existed concerning the small number of studies, lack of objective quantification of food intake, and various definitions used to define active and inactive individuals.

**Conclusion:**

Habitually active individuals showed improved compensation for the energy density of foods, but no consistent differences in appetite or absolute energy intake, in comparison with inactive individuals. This review supports a J-shaped relationship between physical activity level and energy intake. Further studies are required to confirm these findings.

**PROSPERO Registration Number:**

CRD42015019696

**Electronic supplementary material:**

The online version of this article (doi:10.1007/s40279-016-0518-9) contains supplementary material, which is available to authorized users.

## Key Points

**Table:** 

Habitual physical activity and appetite control are not independent of each other; they are interconnected.
The relationship between physical activity level and energy intake is J-shaped.
Objective assessment of all components of energy balance is necessary to improve understanding of this relationship.

## Introduction

The importance of physical activity in reducing morbidity and all-cause mortality [1] and in weight management [2] has become apparent. There has been increasing interest in the relationship between physical activity and appetite control, as both play an integral part in energy balance (e.g. [3–7]). Regular physical activity and exercise training are associated with several physiological adaptations, such as improved insulin sensitivity [8], leptin sensitivity [9, 10], blood pressure [11], blood lipids [12], substrate metabolism [13] and body composition [14], some of which have been proposed as mechanisms involved in eating behaviour [15, 16]. Scientific studies have tended to focus on the appetite responses to exercise rather than habitual physical activity levels per se. This distinction is important to make, as physical activity encompasses occupational, household, transportation and other activities, in addition to structured exercise [17], and the physiological adaptations to exercise and physical activity may differ. Few studies have specifically focused on the appetite control differences between physically active and inactive individuals, but there is some evidence suggesting that habitual physical activity improves appetite control by enhancing satiety signalling [18, 19]. Two recent reviews included secondary analyses on whether the relationship between acute or long-term exercise and energy intake is influenced by physical activity level [20, 21]. From their meta-analysis, Schubert et al. [21] found that absolute energy intake after acute exercise was greater in active individuals than in less active individuals, whereas Donnelly et al. [20] concluded from their systematic review that increased physical activity or exercise, regardless of physical activity level, had no consistent effect on acute or long-term energy intake. However, these reviews included only energy and macronutrient intake as their main outcome measures. As appetite control involves complex co-ordination of a range of homeostatic and non-homeostatic signals in the overall expression of food intake [22], in addition to energy intake, it is important to consider other components, such as appetite-related peptides, subjective appetite sensations, food choice and hedonic reward.

It has been proposed that regulation of the appetite control system and energy intake is improved with increasing levels of physical activity [23]. This issue has yet to be systematically reviewed, and the potential mechanisms behind any improvement in appetite control are unclear. The aims of this systematic review were to examine whether physically active individuals have more sensitive control over appetite than their inactive counterparts and if this confers on them the ability to better match energy intake to energy expenditure, and to identify behavioural or physiological mechanisms underlying any observed differences.

## Methods

This systematic review followed the Preferred Reporting Items for Systematic Reviews and Meta-Analysis (PRISMA) guidelines (Electronic Supplementary Material Appendix S1) and is registered in the PROSPERO database (registration number CRD42015019696).

### Search Strategy

A search was conducted in the databases Ovid Medline, Ovid Embase and SPORTDiscus (EBSCOHost), which included articles published between 1 January 1996 and 15 April 2015, using the strategy (physical activity AND (appetite AND (food intake OR appetite-related peptides))). Previous systematic reviews were screened to identify relevant subject headings and key words to include within each subject category. The specific key words used for the search are listed in Table 1, and the full search strategy for one of the databases that were consulted can be found in Electronic Supplementary Material Appendix S2. Limits were set to include articles published in the English language and studies conducted in human adults aged 18–64 years. Reference lists from the resulting articles were also screened to identify any additional articles.

**Table 1:** Keywords included in database search strategy

Physical activity	Appetite	Food intake	Appetite-related peptides
Motor activity	Appetite	Energy intake	Gut hormone
Exercise	Feeding behaviour	Diet	Gut peptide
Oxygen consumption	Food preferences	Dietary proteins	Peptide YY
Physical fitness	Hunger	Dietary fats	PYY
Exercise tolerance	Satiety	Dietary carbohydrates	Ghrelin
Exercise test	Satiation	Calorie intake	Glucagon-like peptide-1
Physical endurance	Fullness	Food intake	GLP-1
Physical activity	Motivation to eat	Meal size	Pancreatic polypeptide
Physical performance	Food choice	Energy compensation	PP
Aerobic	Food selection	Energy density	Leptin
Aerobic capacity	Desire to eat	Macronutrient	Insulin
Training	Palatability		Cholecystokinin
Maximal VO_2_	Food reward		CCK
Physical capacity	Hedonic		
	Liking		
	Wanting		

*CCK* cholecystokinin, *GLP-1* glucagon-like peptide-1, *PP* pancreatic polypeptide, *PYY* peptide YY, *VO*_*2*_ oxygen consumption

### Study Selection, Inclusion and Exclusion

Articles were included if they involved healthy adults participating in cross-sectional studies and examined appetite control in physically active and inactive individuals. Longitudinal studies assessing appetite control before and after an exercise-training intervention in previously inactive individuals were also included if the intervention lasted greater than 4 weeks (to allow sufficient time for adaptations from regular physical activity to emerge; e.g. see Cornelissen and Smart [11]) and did not include any concurrent dietary intervention (e.g. energy restriction, supplementation). Articles were excluded if they involved animals, children, adolescents, athletes or older adults (>65 years old) and participants who smoked. Abstracts and full-text articles were assessed for eligibility independently by two authors, with uncertainty regarding eligibility being discussed with an additional author.

### Data Extraction and Synthesis

One author extracted the following information into a spreadsheet: authors, date of publication, sample size, participant characteristics (age, sex, body mass index [BMI], percentage body fat, maximal aerobic capacity [VO_2max_], physical activity details), criteria used to assess physical activity status (cross-sectional studies) or training intervention (longitudinal studies), setting, outcome measures (energy intake, appetite ratings and appetite-related peptides) and results. To determine any statistical relationship between habitual physical activity level and energy intake, where data were available, energy intake values were standardized (z-scores) and, from the definitions provided in the studies, physical activity levels were graded into low (<150 min/week, <1000 kcal/week or physical activity level 1.4–1.69), medium (150–419 min/week, 1000–2500 kcal/week or physical activity level 1.7–1.99), high (420–839 min/week or 2500 < 3500 kcal/week) or very high (>840 min/week or ≥3500 kcal/week). One-way analysis of variance (ANOVA) was then used to test for a main effect of graded physical activity level on energy intake score, followed by trend analyses for linear and non-linear functions. Other outcome measures are presented as a qualitative synthesis.

### Risk of Bias

Risk of bias was assessed using the Cochrane Collaboration’s tool for assessing risk of bias for sequence generation, allocation concealment, blinding of participants, personnel and outcome assessors, incomplete outcome data, selective outcome reporting and other sources of bias [24] (Electronic Supplementary Material Table S1). Study inclusion was not influenced by the results of the risk of bias assessment.

## Results

**Fig. 1:**
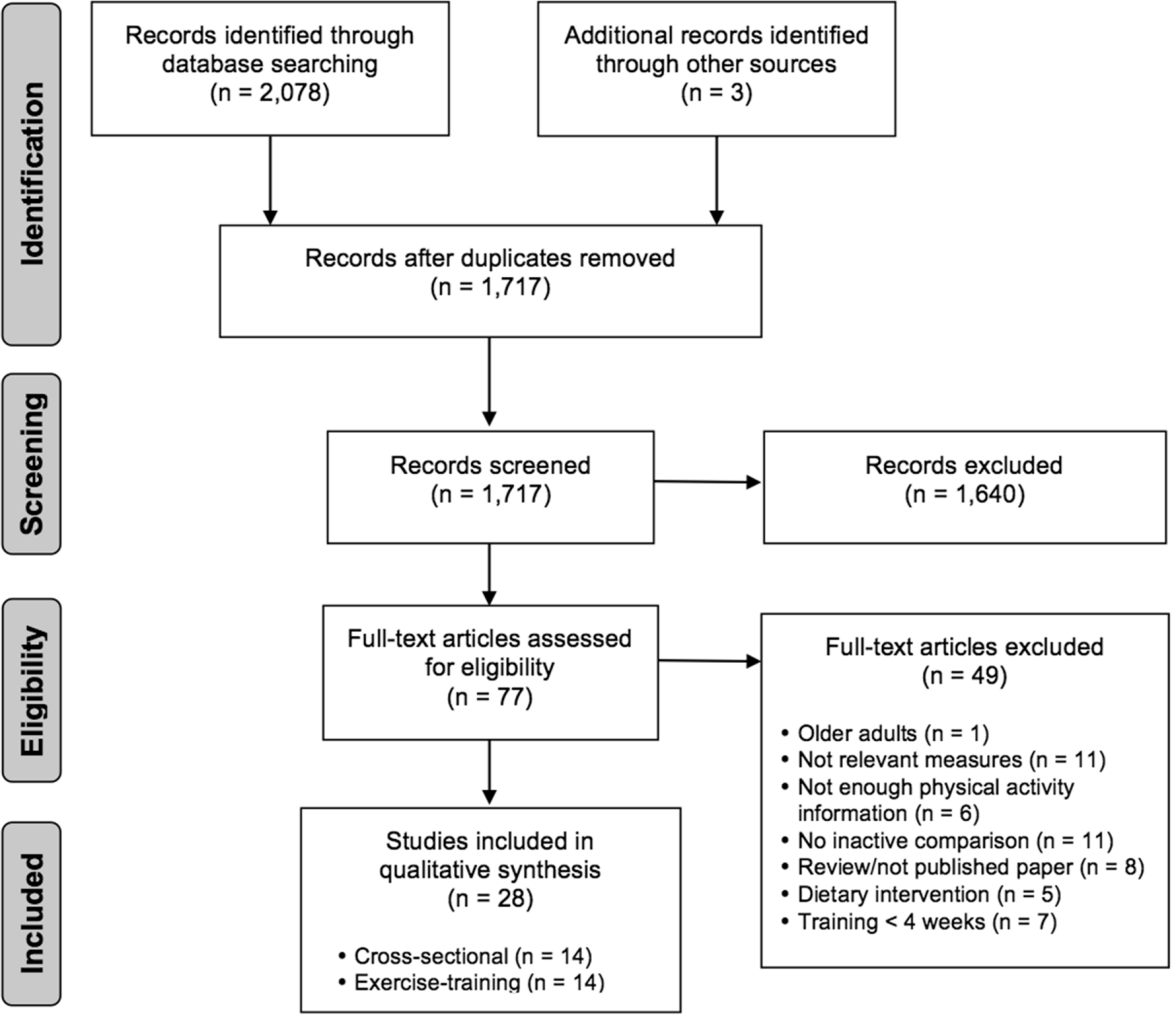
Flow diagram of this systematic review

Figure 1 illustrates the systematic review flow diagram. The database search yielded 2078 articles, 1640 of which were eliminated on the basis of their titles and abstracts alone. The full text was retrieved for 77 articles, and 28 satisfied the inclusion criteria.

### Cross-Sectional Studies

The results from the cross-sectional studies (*n* = 14) are presented in Table 2.

**Table 2:** Cross-sectional studies assessing appetite control in physically active and inactive individuals

Study	Participants	Physical activity status	Setting	Outcome measures	Results
Apolzan et al. [25] Young groups	Men and women Active: *n* = 11 (63.6 % men); age 25 ± 3 years; BMI 23.5 ± 2.0 kg/m^2^; body fat 15.7 ± 6.3 %; VO_2max_ 47.5 ± 6.3 mL/kg/min; PA 2.6 ± 0.7 h/day Inactive: *n* = 13 (61.5 % men); age 25 ± 4 years; BMI 26.6 ± 3.6 kg/m^2^; body fat 23.1 ± 5.0 %; VO_2max_ 33.7 ± 5.8 mL/kg/min; PA 0.0 ± 0.0 h/day	PPAQ and VO_2max_ Active: moderate-intensity PA ≥4 days/week,VO_2max_ above average for age, >2500 kcal/week Inactive: <20 min/day ≤2 days/week, VO_2max_ below average for age, <1000 kcal/week	Free-living	Hunger, fullness, desire to eat (vertical dashes) Food intake (24 h food record)	No effect of activity status on appetite, EI and macronutrient intake
Catenacci et al. [26]	Men and women enrolled in the National Weight Control Registry divided into levels of PA Low: *n* = 910 (21.6 % men); age 49 ± 13 years; BMI 25.8 ± 4.5 kg/m^2^; body fat NR; VO_2max_ NR; PA 416 ± 313 kcal/week Medium: *n* = 934 (21.5 % men); age 48 ± 13 years; BMI 25.2 ± 4.6 kg/m^2^; body fat NR; VO_2max_ NR; PA 1615 ± 355 kcal/week High: *n* = 779 (26.1 % men); age 46 ± 12 years; BMI 24.7 ± 4.7 kg/m^2^; body fat NR; VO_2max_ NR; PA 2256 ± 554 kcal/week Very high: *n* = 968 (27.6 % men); age 44 ± 11 years; BMI 24.5 ± 4.7 kg/m^2^; body fat NR; VO_2max_ NR; PA 5477 ± 2179 kcal/week	PPAQ Low: <1000 kcal/week Medium: 1000 to <2500 kcal/week High: 2500 to <3500 kcal/week Very high: ≥3500 kcal/week	Free-living	Food intake (block FFQ) Restraint, disinhibition and susceptibility to hunger (TFEQ)	No significant differences in EI between groups but higher EI in those reporting the lowest and highest levels of activity (U-shaped relationship with age and sex as covariates) Higher levels of activity had lower % of energy from fat and higher % of energy from carbohydrates Cognitive restraint increased with activity level (linear relationship) No differences in disinhibition and susceptibility to hunger between groups
Charlot and Chapelot [27]	Men High fitness: *n* = 9; age 21 ± 2 years; BMI 23.5 ± 0.7 kg/m^2^; body fat 12.0 ± 2.8 %; VO_2max_ 51.6 ± 6.1 mL/kg/min; PA 8.8 ± 4.5 h/week Low fitness: *n* = 9; age 22 ± 2 years; BMI 26.5 ± 1.3 kg/m^2^; body fat 21.2 ± 2.6 %; VO_2max_ 37.0 ± 5.9 mL/kg/min; PA 2.0 ± 1.8 h/week	VO_2max_ High fitness: VO_2max_ >45 mL/kg/min and >5 h/week of moderate to intense PA Low fitness: VO_2max_ <45 mL/kg/min and <3 h/week of moderate to intense PA	Laboratory and free-living: test meal 60 min after 60 min cycling at 70 % of VO_2max_	Hunger, desire to eat and fullness (VAS) Food intake (1 test meal and food record until breakfast the next day)	No differences in appetite ratings, EI at test meal, macronutrient intake and energy compensation EI from lunch to breakfast and over 24 h significantly greater after exercise compared with resting in both groups
Deshmukh-Taskar et al. [28]	Men and women *n* = 1191 (39.4 % men); age 30 ± 5 years; BMI 27.3 ± 6.7 kg/m^2^; body fat NR; VO_2max_ NR; PA (5-point Likert scale) 3.2 ± 1.1	Answer to “Compared to other people your age and sex, how would you rate your physical activity outside of work during the past year?” from 5-item Likert scale where 1 = physically inactive/sedentary, 3 = moderately active and 5 = very active Active: ≥4 (*n* = 392) Inactive: ≤3 (*n* = 799)	Free-living	Food choices (Youth/Adolescent FFQ)	Active subjects reported greater intake of fruits and 100 % fruit juices and lower intake of burgers and sandwiches than inactive subjects
Georgiou et al. [29]	Men Exercisers: *n* = 89; age 22 ± 2 years; BMI 24.8 ± 4.1 kg/m^2^; body fat NR; VO_2max_ NR; PA NR Non-exercisers: *n* = 51; age 22 ± 2 years; BMI 25.7 ± 5.2 kg/m^2^; body fat NR; VO_2max_ NR; PA NR Women Exercisers: *n* = 106; age 21 ± 2 years; BMI 22.3 ± 3.6 kg/m^2^; body fat NR; VO_2max_ NR; PA NR Non-exercisers: *n* = 73; age 22 ± 2 years; BMI 22.8 ± 4.1 kg/m^2^; body fat NR; VO_2max_ NR; PA NR	Yes or no response to “Do you engage in regular, planned exercise activities in which you work up a sweat, increase your heart rate or breathe faster?”	Free-living	Food choices (National Cancer Institute Health Habits and History FFQ) Health-related influences on food choice questionnaire Perceived change in fat intake	Female and male exercisers considered it more important than non-exercisers to eat the most nutritious foods Female and male exercisers ate more nutrient-dense, low-fat foods than non-exercisers Female exercisers were more likely than non-exercisers to rate 2 % milk, macaroni and cheese, hamburger and peanut butter as fattening Female exercisers reported decreasing intake of high-fat foods (e.g. French fries, cheese and salad dressing) over prior years
Gregersen et al. [30]	Men *n* = 80; age 39 ± 12 years; BMI 25.2 ± 2.7 kg/m^2^; body fat NR; VO_2max_ NR; PA NR Women *n* = 98; age 41 ± 11 years; BMI 24.4 ± 3.0 kg/m^2^; body fat NR; VO_2max_ NR; PA NR	Self-reported PA level (subgroup analysis) High/moderate exercise (*n* = 46): training hard ≥4 h/week Light/no exercise (*n* = 129): light exercise <4 h/week	Laboratory: standardized evening meal to 35 % of individual daily energy requirement	Hunger, fullness, satiety, PFC (VAS) pre and over 3 h post-meal Palatability	Hard/moderate exercisers had lower mean ratings of postprandial satiety and higher mean ratings of post-meal hunger and PFC than light/non-exercisers (differences became non-significant when age and sex were added as covariates) No differences in palatability between groups
Harrington et al. [31]	Non-obese men *n* = 40; age 27 ± 4 years; BMI 23.5 ± 2.5 kg/m^2^; body fat NR; VO_2max_ NR; PA NR Non-obese women *n* = 42; age 27 ± 5 years; BMI 22.4 ± 2.0 kg/m^2^; body fat NR; VO_2max_ NR; PA NR	Activity-related energy expenditure derived from residual value of regression between TDEE from doubly-labelled water and 24 h resting energy expenditure Activity-related energy expenditure divided into low, middle and high tertiles	Laboratory	Food intake (test meal) Hunger, fullness, desire to eat and PFC (VAS) pre and post-test meal SQ	Males in low tertile had a significantly higher fasting desire to eat, PFC and lower fullness than those in the high tertile No differences in fasting appetite between groups in women No differences in appetite ratings after the test meal between groups in both men and women Males in middle tertile had significantly lower EI than high tertile and tended to have lower EI than low tertile Males in high tertile had a significantly lower SQ than the middle tertile for each appetite rating
Jago et al. [32]	Men and women *n* = 1191 (39.3 % men); age 30 ± 5 years; BMI 27.3 ± 6.7 kg/m^2^; body fat NR; VO_2max_ NR; PA (5-point Likert scale) 3.2 ± 1.1	Answer to “Compared to other people your age and sex, how would you rate your physical activity outside of work during the past year?” from 5-item Likert scale where 1 = physically inactive/sedentary, 3 = moderately active and 5 = very active Group 1: *n* = 74; group 2: *n* = 181; group 3: *n* = 544; group 4: *n* = 180, group 5: *n* = 212	Free-living	Food intake (Youth/Adolescent FFQ)	Groups 3, 4 and 5 reported greater intake of dairy products than group 1 Groups 3, 4 and 5 consumed fewer servings of fried foods than group 2 Group 5 had greater EI than group 3, but no differences were seen with the other groups Group 2 consumed greater % of energy from fat than group 4
Jokisch et al. [33]	Men Active: *n* = 10; age 21 ± 2 years; BMI 23.9 ± 1.5 kg/m^2^; body fat 12.6 ± 2.8 %; VO_2max_ NR; PA 438 ± 152 min/week Inactive: *n* = 10; age 21 ± 2 years; BMI 23.0 ± 1.9 kg/m^2^; body fat 15.0 ± 2.3 %; VO_2max_ NR; PA 32 ± 43 min/week	7-day PA recall × 2 Active: ≥150 min/week moderate- to vigorous-intensity PA Inactive: ≤60 min/week moderate- to vigorous-intensity PA	Laboratory: test meal 60 min after 45 min cycling at 65–75 % of HR_max_ or rest	Hunger and liking (VAS) Food intake (1 test meal and food record for remainder of day)	Inactive subjects had greater EI at ad libitum meal after rest than after exercise Both active and inactive subjects had greater EI during remainder of day after exercise compared with rest. Tendency for inactive subjects to have greater EI than active subjects No significant differences in macronutrient intake at test meal, but active subjects consumed greater % of energy from protein than inactive subjects during remainder of day Difference in energy compensation between groups (positive in active and negative in inactive) at test meal, but no differences in energy compensation for remainder of day
Long et al. [19]	Men High exercisers: *n* = 7; age 22 ± 3 years; BMI 22.5 ± 1.5 kg/m^2^; body fat NR; VO_2max_ NR; PA NR Moderate exercisers: *n* = 7; age 27 ± 7 years; BMI 24.1 ± 3.6 kg/m^2^; body fat NR; VO_2max_ NR; PA NR Non-exercisers: *n* = 9; age 22 ± 2 years; BMI 24.1 ± 3.6 kg/m^2^; body fat NR; VO_2max_ NR; PA NR	7-day PA recall × 2 High exercisers: ≥4 exercise sessions/week Moderate exercisers: 2–3 exercise sessions/week Non-exercisers: ≤1 exercise session/week Exercise session: ≥40 min moderate- to high-intensity PA	Laboratory: LE preload and HE preload followed by test meal	Hunger and satiety (VAS) Food intake (1 test meal)	EI in exercisers (groups combined) significantly less after HE versus LE preload EI after HE preload significantly lower in exercisers than in non-exercisers Energy compensation more accurate in active subjects than in inactive subjects Hunger before preload significantly greater in non-exercisers under both HE and LE preloads but no other differences in appetite ratings
Lund et al. [34]	Men Trained: *n* = 10; age 26 ± 3 years; BMI 22 ± 3 kg/m^2^; body fat 12 ± 3 %; VO_2max_ 67 ± 6 mL/kg/min; PA NR Untrained: *n* = 10; age 25 ± 3 years; BMI 22 ± 3 kg/m^2^; body fat 21 ± 3 %; VO_2max_ 42 ± 6 mL/kg/min; PA NR	VO_2max_ Trained: aerobic endurance exercise >3 days/week over several years and VO_2max_ >60 mL/kg/min (runners, cyclists or triathletes) Untrained: no exercise during last 6 months and VO_2max_ <50 mL/kg/min	Laboratory: liquid meal followed by test meal 3 h later	Hunger, satiety, fullness and PFC (VAS) Meal size (test meal) GLP-1, insulin, AG, PYY, PP	GLP-1 and AG higher at baseline in trained subjects GLP-1 higher and insulin lower following liquid meal in trained subjects No group differences in PYY and PP at baseline and in response to liquid meal No group differences in appetite ratings Tendency for greater meal size (in grams) in trained versus untrained subjects, significant after removal of outlier in untrained group
Rocha et al. [35]	Men Active: *n* = 15; age 23 ± 4 years; BMI 22.6 ± 2.0 kg/m^2^; body fat 14.3 ± 3.4 %; VO_2max_ 44.6 ± 5.0 mL/kg/min; PA (TDEE/BMR) 1.80 ± 0.19 Inactive: *n* = 15; age 24 ± 3 years; BMI 25.1 ± 2.4 kg/m^2^; body fat 22.2 ± 3.8 %; VO_2max_ 35.5 ± 5.2 mL/kg/min; PA (TDEE/BMR) 1.54 ± 0.19	Modified Godin Leisure-Time Exercise Questionnaire PA monitor Active: regular exercisers and >150 min/week moderate-intensity PA and PA level 1.70–1.99 Inactive: did not engage in regular exercise and <150 min/week moderate-intensity PA and PA level 1.4–1.69	Laboratory and free-living: test meal following 60 min cycling at 50 % of VO_2max_ or rest	Hunger (VAS) Food intake (1 test meal and food record for remainder of day and subsequent 3 days)	No effects on hunger and EI at test meal Active subjects had greater EI during exercise day than rest day Inactive subjects increased EI on third day after exercise compared with rest Energy compensation observed in active but not inactive subjects during experimental day
Rocha et al. [36]	Women taking oral contraceptives Active: *n* = 10; age 23 ± 4 years; BMI 21.9 ± 1.3 kg/m^2^; body fat 22.5 ± 3.7 %; VO_2max_ 36.8 ± 3.1 mL/kg/min; PA (TDEE/BMR) 1.79 ± 0.13 Inactive: *n* = 10; age 22 ± 3 years; BMI 21.6 ± 2.0 kg/m^2^; body fat 26.7 ± 3.6 %; VO_2max_ 29.9 ± 4.1 mL/kg/min; PA (TDEE/BMR) 1.56 ± 0.15	Modified Godin Leisure-Time Exercise Questionnaire PA monitor Active: regular exercisers and >150 min/week moderate-intensity PA and PA level 1.70–1.99 Inactive: did not engage in regular exercise and <150 min/week moderate-intensity PA and PA level 1.4–1.69	Laboratory and free-living: test meal following 60 min cycling at 50 % of VO_2max_ or rest	Hunger (VAS) Food intake (1 test meal and food record for remainder of day and subsequent 3 days)	No group differences in hunger, EI at test meal and macronutrient intake Inactive subjects had greater EI over the 4 days than active subjects Inactive subjects had lower daily EI the day following exercise compared with rest No energy compensation observed
Van Walleghen et al. [37] Young groups	Men and women Active: *n* = 15 (45.4 % men); age 23 ± 4 years; BMI 23.1 ± 2.7 kg/m^2^; body fat 18.2 ± 8.5 %; VO_2max_ 55.6 ± 10.5 mL/kg/min; PA 575 ± 406 min/week Inactive: *n* = 14 (50 % men); age 26 ± 4 years; BMI 23.5 ± 3.0 kg/m^2^; body fat 27.2 ± 5.6 %; VO_2max_ 37.9 ± 7.1 mL/kg/min; PA 16 ± 37 min/week	Self-reported time spent doing moderate to vigorous PA Active: ≥150 min/week moderate and/or vigorous activity for ≥2 years Inactive: NR	Laboratory and free-living: preload or no preload followed by test meal	Hunger and fullness (VAS) Food intake (1 test meal and food record for remainder of day) Fasting insulin and insulin sensitivity	Active subjects had greater habitual EI, lower % of energy from fat and greater % of energy from carbohydrate than inactive subjects No group differences in appetite and EI at test meal Active subjects had greater EI than inactive subjects during remainder of day in no-preload condition No group differences for energy compensation at test meal, but compensation over the entire day was significantly more accurate in active subjects than in inactive subjects

*AG* acylated ghrelin, *BMI* body mass index, *BMR* basal metabolic rate, *EI* energy intake, *FFQ* food frequency questionnaire, *GLP-1* glucagon-like peptide-1, *HE* high-energy, *HR*_*max*_ maximal heart rate, *LE* low-energy, *NR* not reported, *PA* physical activity, *PFC* prospective food consumption, *PP* pancreatic polypeptide, *PPAQ* Paffenbarger Physical Activity Questionnaire, *PYY* peptide YY, *SQ* satiety quotient, *TDEE* total daily energy expenditure, *TFEQ* Three-Factor Eating Questionnaire, *VAS* visual analogue scale, *VO*_*2max*_ maximal aerobic capacity

#### Study Characteristics: Physical Activity Definitions

The median (range) sample size of the included studies was 15 (7–968) for the active group and 14 (9–910) for the inactive group. Men and women were included in eight studies, of which the median percentage of men was 42.2 % (21.5–63.6 %) in the active group and 50 % (21.6–61.6 %) in the inactive group [25, 26, 28–32, 37]. Five studies included only men [19, 27, 33–35], and one study included only women [36].

Physical activity status was determined by self-report (a physical activity questionnaire, physical activity level question or physical activity recall) in 11 studies [19, 26, 28–30, 32, 33, 37], by a VO_2max_ test in three studies [25, 27, 34] or from total daily energy expenditure (TDEE) and resting energy expenditure or basal metabolic rate (BMR) in three studies [31, 35, 36]. Only three studies used a combination of self-reported and objectively measured physical activity status [25, 35, 36].

The active groups were defined as participating in moderate to vigorous physical activity for at least 150 min/week [33, 35–37], 4 h/week [30], 5 h/week with a VO_2max_ greater than 45 mL/kg/min [27], 3 days/week with a VO_2max_ greater than 60 mL/kg/min [34], 4 days/week and >2500 kcal/week with a VO_2max_ above average for age [25], or 1000 kcal/week [26]. A TDEE/BMR value between 1.70 and 1.99 was utilized in two studies [35, 36]. Moderate exercisers participated in 2–3 sessions/week of at least 40 min of moderate- to high-intensity physical activity [19] or expended between 1000 and 2500 kcal/week [26]. High exercisers participated in four or more structured exercise sessions/week of at least 40 min of moderate- to high-intensity physical activity [19] or expended 2500 > 3500 kcal/week [26], whereas very high exercisers expended 3500 kcal/week or greater [26].

The inactive groups were defined as having not exercised over the previous 6 months and VO_2max_ values less than 50 mL/kg/min [34] or less than 1 session/week of moderate- to high-intensity physical activity [19], 20 min/day and 2 days/week [25], 60 min/week [33], 1000 kcal/week [26], 150 min/week of moderate-intensity physical activity [35, 36], 3 h/week of moderate- to high-intensity physical activity with a VO_2max_ less than 45 mL/kg/min [27] or 4 h/week [30]. Two studies used a TDEE/BMR value between 1.4 and 1.69 [35, 36].

On the basis of the physical activity definitions above, for the purposes of statistical treatment, we distinguished physical activity levels as low (<150 min/week, <1000 kcal/week or physical activity level 1.4–1.69), medium (150–419 min/week, 1000–2500 kcal/week or physical activity level 1.7–1.99), high (420–839 min/week or 2500 > 3500 kcal/week) and very high (>840 min/week or ≥3500 kcal/week) for analysis of standardized energy intake.

#### Study Characteristics: Appetite-Related Measures

Five studies evaluated appetite measures in a laboratory [19, 30, 31, 33, 34], five studies did so in free-living conditions [25, 26, 28, 29, 32] and four studies combined laboratory and free-living measures [27, 35–37]. Four studies included exercise (45–60 min cycling at 50–75 % of VO_2max_ or maximal heart rate [HR_max_]) during the laboratory session [27, 33, 35, 36]. Ten studies included fasting and/or daily (area under the curve) subjective appetite ratings, all of which included hunger [19, 25, 27, 30, 31, 33–37]. Other appetite ratings assessed were fullness [25, 27, 30, 31, 33, 34, 37], prospective food consumption (PFC) [30, 31, 34], desire to eat [25, 27, 31], satiety [19, 30, 34], liking [33] and palatability [30]. One study reported restraint, disinhibition and susceptibility to hunger [26]. Eleven studies assessed energy intake, via either a food frequency questionnaire (FFQ) [26, 32], a food record [25], laboratory-based test meals [19, 31, 34] or a combination of laboratory-based test meals and food records [27, 33, 35–37]. Six studies reported energy compensation following either a preload [19, 37] or a single bout of exercise [27, 33, 35, 36]. Eight studies reported macronutrient intake [25–27, 32, 33, 35–37]. Three studies assessed food choices via an FFQ [28, 29, 32]. Two studies included assessment of appetite-related peptides [34, 37].

#### Participant Characteristics

The median (range) age was 23 (21–48) years for the active group and 22 (21–49) years for the inactive group.

In the ten studies that reported BMI for the active and inactive groups separately, the median (range) was 23.5 (21.9–25.2) kg/m^2^ for the active group and 24.1 (21.6–26.6) kg/m^2^ for the inactive group [19, 25–27, 29, 33–37]. In three studies, the inactive group had a significantly greater BMI than the active group [25, 27, 35]. In the studies that reported BMI for the groups combined, the median (range) was 24.8 (22.4–27.3) kg/m^2^ [28, 30–32].

In the seven studies that reported percentage body fat, the median (range) was 14.3 (12.0–22.5) % for the active group and 22.2 (15.0–27.2) % for the inactive group [25, 27, 33–37]. In all studies, the inactive group had a significantly greater percentage body fat than the active group.

In the six studies that reported VO_2max_, the median (range) was 49.6 (36.8–67.0) mL/kg/min for the active group and 36.3 (29.9–42.0) mL/kg/min for the inactive group [25, 27, 34–37]. In all studies, the active group had a significantly greater VO_2max_ than the inactive group.

#### Study Results: Appetite Ratings

Of the ten studies that measured appetite ratings, three found differences between the physically active and inactive groups. Harrington et al. [31] reported greater fasting appetite and lower satiety quotient (SQ) [calculated as (pre-meal appetite rating minus post-meal appetite rating) divided by energy intake] for hunger, fullness, desire to eat and PFC in men in the high activity tertile compared to the moderate activity tertile, whereas Long et al. [19] reported greater fasting appetite in the inactive group. Gregersen et al. [30] found greater postprandial appetite in the active group, however differences became non-significant when age and sex were added as covariates.

#### Study Results: Energy and Macronutrient Intake

Ten of 11 studies found differences in energy intake between active and inactive individuals. Two studies found greater energy intake (habitual energy intake [37] or with a test meal [34]) in the active compared to the inactive group, whereas one study observed greater energy intake in inactive women over 4 days than active women [36]. Furthermore, two studies observed a non-linear relationship in energy intake, whereby energy intake was highest in the groups with the lowest and highest levels of physical activity [26, 31], while Jago et al. [32] only observed a greater energy intake in the very active group compared to the moderately active group. In studies assessing energy intake following a preload, Long et al. [19] found that energy intake at an ad libitum test meal following a high-energy preload was significantly lower than following the low-energy preload in regular exercisers. The same study showed that compared to non-exercisers, energy intake following the high-energy preload was significantly lower in exercisers. Moreover, Van Walleghen et al. [37] found that the active group consumed more throughout the day following the no-preload condition than the inactive group, leading to significantly more accurate short-term energy compensation. Of note, however, there were no differences in energy compensation between groups at the test meal after the preload [37]. In studies measuring energy intake after exercise, two of three studies in men observed energy compensation in the active group, where energy intake following an exercise session was greater compared to rest at test meal [33] or throughout the day (but not at the test meal in this study) [35]. One of these studies observed negative energy compensation in the inactive group, where energy intake was lower following the exercise session compared to rest, suggesting an effect of exercise-induced anorexia [33]. Of the above studies that observed differences between groups, only four were based on objectively measured (test meal) energy intake [19, 31, 33, 34].

As for macronutrient intake, compared to the inactive group, two studies found that the active group consumed a greater percentage of energy from carbohydrates [26, 37], three found a lower percentage of energy from fat [26, 32, 37], while one study found a greater percentage of energy from protein [33]. In terms of food choices, active individuals reported a greater intake of nutrient-dense, low-fat foods [29], fruits and 100 % fruit juices [28], and dairy products [32], and a lower intake of burgers and sandwiches [28] and fried foods [32] than inactive.

#### Study Results: Standardized Energy Intake

**Fig. 2:**
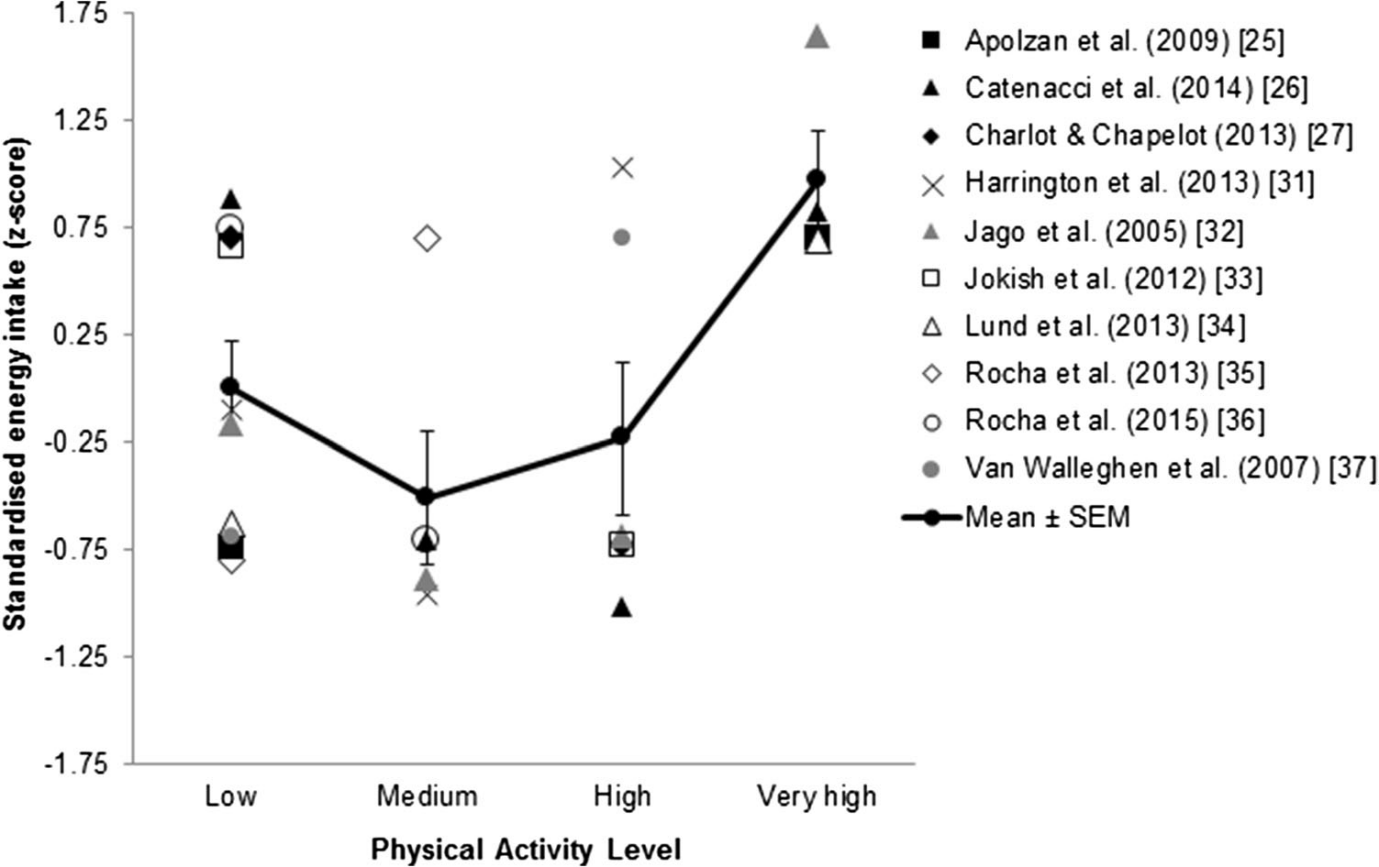
Standardized energy intake by physical activity level from the ten cross-sectional studies reporting energy intake (*n* = 25 data points). Trend analysis confirmed significant linear (*P* < 0.05) and quadratic (*P* < 0.01) relationships between the graded physical activity level and energy intake scores. The *thick black line* indicates the mean of the z-scores. *SEM* standard error of the mean

To further examine the relationship between energy intake and physical activity level, the available energy intake data from the cross-sectional studies [25–27, 31–37] were extracted and transformed into standardized scores then plotted according to physical activity level (low, medium, high, very high) as described in Sect. 3.1.1. In the studies that included a preload or an exercise bout [27, 33, 35, 36], energy intake was taken from the control condition. Of these ten studies, eight were based on self-reported daily energy intake [25–27, 32, 33, 35–37] while two were based on energy intake at a test meal [31, 34]. The pattern of means revealed a J-shaped curve for energy intake as habitual physical activity level increased (Fig. 2). One-way ANOVA confirmed a main effect of graded physical activity level on energy intake score [*F*(3,21) = 3.57, *P* = 0.03]. Post hoc trend analyses revealed significant effects for linear [*F* = 5.79, *P* = 0.03] and curvilinear (quadratic) [*F* = 8.10, *P* = 0.01] functions.

#### Study Results: Appetite-Related Peptides

Van Walleghen et al. [37] found greater insulin sensitivity in the active group. Lund et al. [34] found that in active individuals, glucagon-like peptide-1 (GLP-1) and acylated ghrelin were higher at baseline (insulin tended to be lower), and following a liquid meal, GLP-1 was higher and insulin was lower in active. No group differences were found for peptide YY (PYY) and pancreatic polypeptide.

### Exercise-Training Interventions

The results from the exercise-training interventions (*n* = 14) are presented in Table 3.

**Table 3:** Studies investigating the effect of exercise training on appetite control in previously inactive individuals

Study	Participants	Training intervention	Setting	Outcome measures	Results
Alkahtani et al. [38]	Overweight and obese men *n* = 10; age 29 ± 4 years; BMI baseline 30.7 ± 3.4 kg/m^2^; BMI post NR; body fat baseline 31.2 ± 4.7 %; body fat post NR; VO_2max_ baseline 28.7 ± 3.4 mL/kg/min; VO_2max_ post NR	4 weeks supervised MIIT 3 days/week (30–45 min of 5 min stages at ±20 % workload at 45 % of VO_2peak_) 4 weeks supervised HIIT 3 days/week (30–45 min of 30 s at 90 % of VO_2peak_ and 30 s rest) Each training block was counterbalanced and separated by a 6-week detraining washout	Laboratory: test meal following 45 min cycling at 45 % of VO_2max_ pre and post both training blocks	Hunger, desire to eat and fullness (VAS) Liking and wanting (computer-based paradigm) Food intake (test meal)	Tendency for suppression of desire to eat after acute exercise post-training with HIIT compared with MIIT Tendency for explicit liking for high-fat, non-sweet foods after acute exercise to increase with MIIT and decrease with HIIT post-training No effects of training on food intake and EI Tendency for fat intake and % of energy from fat to increase after MIIT
Bryant et al. [39]	Overweight and obese men and women *n* = 58 (32.7 % men); age 36 ± 10 years; BMI baseline 31.8 ± 4.5 kg/m^2^; BMI post 30.7 ± 4.4 kg/m^2^; body fat baseline 34.8 ± 7.8 %; body fat post 31.9 ± 9.0 %; VO_2max_ baseline 29.1 ± 5.7 mL/kg/min; VO_2max_ post NR	12 weeks supervised aerobic exercise 5 days/week (500 kcal at 70 % of HR_max_)	Laboratory	Food intake (self-determined fixed breakfast followed by 2 ad libitum meals and evening snack box) Restraint, disinhibition and susceptibility to hunger (TFEQ)	No change in 24 h EI and susceptibility to hunger Significant reduction in disinhibition and increase in restraint after training
Caudwell et al. [40]	Overweight and obese men *n* = 14; age 44 ± 6 years; BMI baseline 31.3 ± 5.0 kg/m^2^; BMI post 30.5 ± 4.9 kg/m^2^; body fat baseline 34.3 ± 7.0 %; body fat post 32.4 ± 7.6 %; VO_2max_ NR Overweight and obese premenopausal women *n* = 27; age 42 ± 8 years; BMI baseline 30.4 ± 3.2 kg/m^2^; BMI post 30.2 ± 3.6 kg/m^2^; body fat baseline 44.0 ± 5.5 %; body fat post 42.5 ± 5.8 %; VO_2max_ NR	12 weeks supervised aerobic exercise 5 days/week (500 kcal at 70 % of HR_max_)	Laboratory: HE and LE density probe days	Food intake (self-determined fixed breakfast, fixed energy lunch and ad libitum dinner and evening snack box)	Significant effect of training on HE density meal size but not LE density meal size No effect of training on daily EI under each dietary condition
Caudwell et al. [41]	Overweight and obese men *n* = 35; age 41 ± 9 years; BMI baseline 30.5 ± 8.6 kg/m^2^; BMI post 29.6 ± 1.1 kg/m^2^; body fat baseline 33.8 ± 6.6 %; body fat post 31.3 ± 3.3 %; VO_2max_ baseline 34.9 ± 6.9 mL/kg/min, VO_2max_ post 43.3 ± 6.9 mL/kg/min Overweight and obese premenopausal women *n* = 72; age 41 ± 10 years; BMI baseline 31.8 ± 4.3 kg/m^2^; BMI post 30.9 ± 1.1 kg/m^2^; body fat baseline 44.1 ± 6.0 %; body fat post 41.6 ± 2.2 %; VO_2max_ baseline 29.1 ± 6.5 mL/kg/min; VO_2max_ post 35.1 ± 5.5 mL/kg/min	12 weeks supervised aerobic exercise 5 days/week (500 kcal at 70 % of HR_max_)	Laboratory	Hunger, fullness and desire to eat (VAS) SQ Food intake (self-determined fixed breakfast, fixed energy lunch and ad libitum dinner and evening snack box)	No change in 24 h EI with training Significant increase in fasting hunger but no change in daily hunger AUC SQ significantly greater post-training
Cornier et al. [42]	Overweight and obese men and women *n* = 12 (41.6 % men); age 38 ± 10 years; BMI baseline 33.3 ± 4.3 kg/m^2^; BMI post NR; body fat baseline 36.5 ± 1.9 %; body fat post 34.4 ± 2.0 %; VO_2max_ NR	6 months supervised treadmill walking 5 days/week (building up to 500 kcal/day at 75 % of VO_2max_)	Laboratory and free-living: test meal breakfast (30 % of estimated daily energy requirements)	Leptin Restraint and disinhibition (TFEQ) Power of Food Scale Craving and Mood Questionnaire Food Craving Inventory Hunger, satiety and PFC (VAS) Food intake (3-day food record)	Significant reduction in fasting leptin post-training No change in dietary restraint or disinhibition, food cravings, Power of Food Scale, food desire and appeal, or postprandial appetite ratings Self-reported EI lower after training compared with baseline but no change in macronutrient intake
Guelfi et al. [43] Exercise groups	Overweight and obese men (age 49 ± 7 years) Aerobic training: *n* = 12; BMI baseline 31.7 ± 3.5 kg/m^2^; BMI post 31.1 ± 3.3 kg/m^2^; body fat NR; VO_2max_ baseline 2.25 ± 0.51 L/min at 80 % of HR_max_; VO_2max_ post 2.82 ± 0.60 L/min at 80 % of HR_max_ Resistance training: *n* = 13; BMI baseline 30.3 ± 3.5 kg/m^2^; BMI post 30.3 ± 3.7 kg/m^2^; body fat NR; VO_2max_ baseline 1.94 ± 0.39 L/min at 80 % of HR_max_; VO_2max_ post 2.17 ± 0.54 L/min at 80 % of HR_max_	12 weeks supervised (3 days/week) aerobic exercise (40–60 min at 70–80 % of HR_max_) or resistance exercise (weight training matched for duration and intensity; 3–4 sets of 8–10 repetitions of 9 exercises at 75–85 % of 1RM)	Laboratory: 2 h, 75 g OGTT	Hunger and fullness (VAS) Active ghrelin, leptin, insulin, insulin sensitivity, PP and PYY	Significant increase in fasting and postprandial fullness following aerobic training only No change in fasting or postprandial hunger with training Fasting and postprandial leptin were significantly lower after training Postprandial insulin was significantly lower after aerobic training only No change in fasting insulin, and fasting and postprandial AG, PP and PYY post-training Improvement in insulin sensitivity in both groups post-training
Jakicic et al. [44] Exercise groups	Overweight women Moderate dose: *n* = 76; age 44 ± 8 years; BMI baseline 27.2 ± 1.8 kg/m^2^; BMI post 26.9 ± 2.1 kg/m^2^; body fat baseline 33.5 ± 4.1 %; body fat post 33.3 ± 4.8 %; VO_2max_ NR High dose: *n* = 88; age 46 ± 8 years; BMI baseline 27.0 ± 1.6 kg/m^2^; BMI post 26.7 ± 2.4 kg/m^2^; body fat baseline 33.0 ± 4.1 %; body fat post 32.3 ± 5.3 %; VO_2max_ NR	18 months unsupervised moderate dose (150 min/week) or high dose (300 min/week) exercise ~5 days/week in bouts ≥10 min at moderate to vigorous intensity (55–85 % of HR_max_)	Free-living	Food intake (FFQ) Eating Behaviour Inventory	No group-by-time interaction on EI and macronutrient intake Eating behaviour score improved post-intervention but no differences between groups
King et al. [45]	Overweight and obese men and women Compensators: *n* = 18 (23.5 % men); age 38 ± 9 years; BMI baseline 30.7 ± 2.9 kg/m^2^; BMI post NR; body fat baseline 32.7 ± 8.0 %; body fat post NR; VO_2max_ baseline 28.8 ± 5.7 mL/kg/min; VO_2max_ post NR Non-compensators: *n* = 17 (33.3 % men); age 40 ± 13 years; BMI baseline 33.1 ± 4.7 kg/m^2^; BMI post NR; body fat baseline 37.2 ± 7.9 %; body fat post NR; VO_2max_ baseline 28.4 ± 5.8 mL/kg/min; VO_2max_ post NR	12 weeks supervised aerobic exercise 5 days/week (500 kcal at 70 % of HR_max_)	Laboratory	Hunger, fullness, PFC and desire to eat (VAS) Food intake (self-determined fixed breakfast followed by 2 ad libitum meals and evening snack box)	No significant changes in 24 h EI in pooled data with training; however, compensators increased EI and % of energy from fat, and non-compensators decreased EI from baseline to post-intervention Compensators had greater hunger profile post-training than non-compensators
King et al. [18]	Overweight and obese men and women divided into responders (*n* = 32) and non-responders (*n* = 26) to exercise-induced weight loss *n* = 58 (32.7 % men); age 40 ± 10 years; BMI baseline 31.8 ± 4.5 kg/m^2^; BMI post NR; body fat NR; VO_2max_ baseline 29.1 ± 5.7 mL/kg/min; VO_2max_ post NR	12 weeks supervised aerobic exercise 5 days/week (500 kcal at 70 % of HR_max_)	Laboratory: self-determined fixed breakfast	Hunger, fullness, PFC and desire to eat (VAS), SQ	Non-responders and responders had significantly greater fasting hunger but also a greater SQ post-training Only non-responders increased daily motivation to eat (greater hunger, desire to eat and lower fullness) post-training
Martins et al. [46]	Men and women *n* = 25 (44 % men); age 30 ± 12 years; BMI baseline 22.7 ± 2.3 kg/m^2^; BMI post 22.8 ± 2.2 kg/m^2^; body fat baseline 23.6 ± 7.8 %; body fat post 23.0 ± 7.5 %; VO_2max_ baseline 31.1 ± 4.8 mL/kg/min; VO_2max_ post 34.3 ± 7.4 mL/kg/min	6 weeks unsupervised aerobic exercise ≥4 days/week, 30–45 min (continuously or bouts ≥10 min each) at 65–75 % of HR_max_	Laboratory and free-living: LE preload and HE preload	Hunger, fullness, palatability (VAS) Food intake (1 test meal and food record until breakfast next morning) Fasting insulin and insulin sensitivity	Test meal size and cumulative 24 h EI significantly lower following HE preload versus LE preload post-training No improvement in energy compensation at test meal but tendency for improved compensation over 24 h Greater % of energy from protein at test meal after training No change in fasting insulin and insulin sensitivity No change in appetite ratings
Martins et al. [47]	Overweight and obese men and women *n* = 15 (53.3 % men); age 37 ± 8 years; BMI baseline 31.3 ± 2.3 kg/m^2^; BMI post 30.1 ± 2.3 kg/m^2^; body fat baseline 35.3 ± 5.6 %; body fat post 33.5 ± 5.9 %; VO_2max_ baseline 32.9 ± 6.6 mL/kg/min; VO_2max_ post 37.7 ± 5.9 mL/kg/min	12 weeks supervised aerobic exercise 5 days/week (500 kcal at 75 % of HR_max_)	Laboratory: standardized breakfast	Hunger, fullness, PFC and desire to eat (VAS), AG, TG, insulin, insulin sensitivity, GLP-1, PYY over 3 h post-breakfast	Significant reduction in fasting and postprandial insulin post-training Improvement in insulin sensitivity post-training Increase in fasting AG after training but no change in postprandial AG No significant training effect on TG, GLP-1 and PYY, but tendency for greater GLP-1 AUC in late postprandial period after training Significant increases in fasting hunger, desire to eat and PFC, and decrease in fullness post-training Greater postprandial hunger and desire to eat post-training
Martins et al. [48]	Overweight and obese men and women *n* = 15 (53.3 % men); age 37 ± 8 years; BMI baseline 31.3 ± 2.3 kg/m^2^; BMI post 30.1 ± 2.3 kg/m^2^; body fat baseline 35.3 ± 5.6 %; body fat post 33.5 ± 5.9 %; VO_2max_ baseline 32.9 ± 6.6 mL/kg/min; VO_2max_ post 37.7 ± 5.9 mL/kg/min	12 weeks supervised aerobic exercise 5 days/week (500 kcal at 75 % of HR_max_)	Laboratory and free-living: (1) standardized breakfast (2) LE preload (3) HE preload	Hunger, fullness, PFC and desire to eat (VAS) Food intake (1 test meal after preload and food record for remainder of day) CCK and leptin over 3 h post-breakfast	Significant reduction in fasting and postprandial leptin post-training but no change in CCK No change in test meal EI but cumulative EI after HE preload significantly lower than LE preload post-training, whereas it was greater than LE at baseline Greater accuracy in energy compensation post-training No change in macronutrient intake No effect of training on appetite ratings after preloads
Rosenkilde et al. [49] Exercise groups	Overweight men Moderate-dose group: *n* = 18; age 30 ± 7 years; BMI baseline 28.6 ± 1.8 kg/m^2^; BMI post 27.5 ± 2.0 kg/m^2^; body fat NR; VO_2max_ baseline 34.6 ± 24.1 mL/kg/min; VO_2max_ post 42.3 ± 4.5 mL/kg/min High-dose group: *n* = 18; age 28 ± 5 years; BMI baseline 27.6 ± 1.4 kg/m^2^; BMI post 26.9 ± 1.2 kg/m^2^; body fat NR; VO_2max_ baseline 36.2 ± 5.3 mL/kg/min; VO_2max_ post 43.1 ± 6.6 mL/kg/min	12 weeks unsupervised daily endurance exercise expending 300 kcal/day (moderate dose) or 600 kcal/day (high dose) at >50 % of VO_2max_	Laboratory: (1) standardized breakfast (2) exercise test (1 h ~60 % of VO_2max_)	Hunger, satiety, fullness, PFC, palatability and liking (VAS) Food intake (lunch test meal after breakfast) Restraint, disinhibition and susceptibility to hunger (TFEQ) Insulin, PYY_3-36_, and ghrelin post-breakfast	Fasting and postprandial AUC for insulin significantly lower after both exercise interventions No training effect on PYY_3-36_ and ghrelin Fasting and postprandial fullness increased in high-dose group post-intervention No differences in EI, palatability, liking, restraint, disinhibition and susceptibility to hunger within groups
Shaw et al. [50] Exercise group	Men *n* = 13; age 28 ± 5 years; BMI NR; body fat baseline 26.8 ± 1.5 %; body fat post 23.3 ± 6.3 %; VO_2max_ NR	8 weeks supervised resistance exercise 3 days/week (3 sets of 15 repetitions of 9 exercises)	Free-living	Food intake (3-day food record)	No change in EI and macronutrient intake with training

*1RM* 1 repetition maximum, *AG* acylated ghrelin, *AUC* area under the curve, *BMI* body mass index, *CCK* cholecystokinin, *EI* energy intake, *FFQ* food frequency questionnaire, *GLP-1* glucagon-like peptide-1 *HE* high-energy, *HIIT* high-intensity interval training, *HR*_*max*_ maximal heart rate, *LE* low-energy, *MIIT* moderate-intensity interval training, *NR* not reported, *OGTT* oral glucose tolerance test, *PFC* prospective food consumption, *PP* pancreatic polypeptide, *PYY* peptide YY, *PYY*_*3-36*_ peptide YY (3-36), *SQ* satiety quotient, *TFEQ* Three-Factor Eating Questionnaire, *TG* total ghrelin, *VAS* visual analogue scale, *VO*_*2max*_ maximal aerobic capacity, *VO*_*2peak*_ peak aerobic capacity

#### Study Characteristics: Exercise Intervention

The median (range) duration of the interventions was 12 (4–72) weeks of exercise 5 (3–7) days/week. Exercise duration was prescribed in minutes or energy expenditure (kcal), at intensities in percentage of VO_2max_ or percentage of HR_max_. The median exercise prescription was 43.8 (30–60) min or 500 (300–600) kcal/session at 68.5 (45–90) % of VO_2max_ or 70 (70–75) % of HR_max_. Eleven training interventions involved aerobic exercise [18, 39–43, 45–49], two interventions involved resistance exercise [43, 50] and one intervention compared moderate-intensity interval training and high-intensity interval training in a crossover design [38]. One study did not specify the exercise modality [44]. In 11 of the 14 interventions the exercise was supervised [18, 39–43, 45, 47, 48]. Nine studies collected appetite-related measures in a laboratory [18, 38–41, 43, 45, 47, 49], two studies in free-living conditions [44, 50], and three studies in a combination of laboratory and free-living conditions [42, 46, 48].

#### Study Characteristics: Appetite-Related Measures

Ten studies included fasting and/or daily (area under the curve) appetite ratings, all of which included hunger [18, 38, 40, 42, 43, 45–49]. Fullness [18, 38, 41, 43, 45–49], PFC [18, 42, 45, 47–49], desire to eat [18, 38, 41, 45, 47, 48], satiety [42, 49], liking and palatability [46, 49] were also assessed. Three studies measured restraint, disinhibition and susceptibility to hunger [39, 42, 49]; one study included the Power of Food Scale, the Craving and Mood Questionnaire and the Food Craving Inventory [42]; one study included the Eating Behaviour Inventory [44]; and one study assessed liking and wanting for foods varying in fat and sweetness [38]. Eleven studies assessed energy intake, via an FFQ [44], food record [42, 50], test meals [38–41, 45, 49], or combination of test meals and food records [46, 48]. Two studies measured energy intake following high- and low-energy preloads [46, 48] and one at high- and low-energy density meals [40]. Seven studies reported macronutrient intake [38, 42, 44–46, 48, 50]. Six studies assessed appetite-related peptides in the fasting state [42, 43, 46–49] and three in response to food ingestion [43, 47, 48].

#### Participant Characteristics

The median (range) age was 38 (28–49) years and the sample size of the included studies was 18 (10–88). Men and women were included in nine studies, of which the median percentage of men was 33.7 (23.5–53.3) % [18, 39–42, 45–48]. Four studies only included men [38, 43, 49, 50] and one study only included women [44].

Nine studies reported BMI before and after the intervention [39–41, 43, 44, 46–49], the median (range) was 30.5 (22.7–31.8) kg/m^2^ at baseline and 30.1 (22.8–31.1) kg/m^2^ post-intervention. Seven of these reported a significantly lower BMI after the exercise intervention [39, 41, 43, 44, 47–49]. In the four studies that only reported baseline BMI [18, 38, 42, 45], the median (range) was 31.8 (30.7–33.3) kg/m^2^.

Eight studies reported percentage body fat values before and after the intervention, the median (range) was 34.3 (23.6–44.1) % at baseline and 32.4 (23.0–42.5) % post-intervention [39–41, 44, 46–48, 50]. Seven of these reported a significantly lower percentage body fat after the intervention [39–41, 44, 47, 48, 50]. In the three studies that reported only baseline percentage body fat, the median (range) was 34.6 (31.2–37.2) % [38, 42, 45].

In the five studies that reported VO_2max_ before and after the intervention, the median (range) was 32.9 (29.1–36.2) mL/kg/min at baseline and 37.7 (34.3–43.3) mL/kg/min post-intervention [41, 46–49]. In all studies, the increase in VO_2max_ with training was significant. In the four studies that only reported baseline VO_2max_, the median (range) was 28.8 (28.4–29.1) mL/kg/min [18, 38, 39, 45].

#### Study Results: Appetite Ratings

Exercise training led to differences in appetite ratings in five of ten studies. Three studies found an increase in fasting hunger [18, 41, 47], desire to eat and PFC [47], and a decrease in fullness [47]. However, two studies found that fasting fullness increased following aerobic [43] and high-dose aerobic (600 kcal/day) [49] exercise training. King et al. [18] reported a greater daily hunger, desire to eat and lower fullness post-training in a subsample of non-responders to exercise-induced weight loss (i.e. individuals with changes in body composition below that expected based on the total exercise-induced energy expenditure). In response to a standardized breakfast, Martins et al. [47] found an increase in hunger and desire to eat following exercise training, whereas Guelfi et al. [43] found an increase in fullness after an oral glucose tolerance test following aerobic training.

The two studies that included the SQ found increases post-training [18, 41]. Only one of three studies found a reduction in disinhibition and an increase in restraint post-training [39].

#### Study Results: Energy and Macronutrient Intake

Five of 11 studies found differences in energy intake after the exercise-training intervention. Daily energy intake was lower post-training in one study [42], while it increased in a subsample of compensators in another study [45]. As for high-energy test meal challenges, Caudwell et al. [40] showed a reduction in meal size containing high-energy density foods, and two studies demonstrated that energy intake was lower throughout the day after a high-energy preload compared to a low-energy preload [46, 48].

One study showed an increase in the percentage of energy from fat in a subsample of compensators (individuals whose weight loss after exercise training was less than predicted on the basis of the total exercise-induced energy expenditure) [45] and another after moderate-intensity interval training [38]. Training led to an increase in the percentage of energy from protein in another study [46].

#### Study Results: Appetite-Related Peptides

Of the studies that assessed fasting peptides, five found differences following exercise training, where leptin [42, 43, 48] and insulin decreased [47, 49], and ghrelin increased [47]. Insulin sensitivity improved after training in two of three studies [43, 47]. Of note, the study that found no improvement in insulin sensitivity was half the duration of the two others (6 vs 12 weeks) [46]. All three studies that assessed the peptide response to food ingestion found training effects, where postprandial leptin [43, 48] and insulin decreased [43, 47] after aerobic training, while there was a tendency for GLP-1 in the late postprandial period to increase with training [47].

## Discussion

### Appetite Control in Active and Inactive Individuals

This systematic review investigated differences in appetite ratings, food intake and appetite-related peptides between active and inactive (or previously inactive) individuals in order to determine whether habitual physical activity improves appetite control. In terms of fasting, postprandial or daily appetite ratings, studies reported mixed results, such that no clear differences could be distinguished between physically active and inactive individuals. It has been suggested that combining appetite sensations with objectively measured energy intake to calculate parameters such as the SQ can provide a better indication of the ability of the energy consumed to affect appetite. One cross-sectional study [31] and two exercise-training studies [18, 41] assessed the SQ, with conflicting results; however, the former measured the SQ during an ad libitum meal while in the latter studies, the SQ was measured during a standardized meal. These differences, along with differences in the protocols in the other studies, may have accounted for the contradictory results in appetite ratings.

Several studies focused on the measurement of energy intake, but, again, no consistent differences were found between active and inactive individuals. However, these simple comparisons precluded the possibility that physical inactivity may lead to a dysregulation of appetite and subsequent overconsumption, meaning that differences between active and inactive individuals may not always be apparent. Indeed, we have recently argued that the relationship between physical activity level and energy intake may follow a curvilinear function [23]. After transforming absolute energy intake into standardized scores and distinguishing levels of physical activity from the definitions of the ‘active’ groups used in the cross-sectional studies, we were able to test this hypothesis. The results revealed a significant quadratic effect illustrated by a J-shaped curve across physical activity levels (see Fig. 2). A similar J-shaped relationship has recently been suggested by Shook et al. [51], who compared estimated energy intake, using an equation based on changes in body composition, across quintiles of physical activity in a large heterogeneous sample of young adults. Their analysis provides further support to our synthesis of the literature, which demonstrates that the relationship between physical activity level and energy intake is non-linear, as was postulated by Mayer et al. [52] almost 60 years ago. In Bengali jute mill workers whose daily occupations ranged from ‘sedentary’ to ‘very heavy’ work, daily energy expenditure and daily energy intake were closely matched at higher levels of daily physical activity, but at low levels of daily physical activity, this coupling was lost, such that daily energy intake exceeded expenditure in those performing ‘sedentary’ or ‘light’ work [52]. This relationship may explain why differences in energy intake may not be obvious between active and inactive individuals, as they stand at similar levels on the energy intake curve. As our findings are based on standardized scores from the results of studies using various methodologies and protocols [25–27, 31–37], and Shook et al. [51] inferred from changes in body composition, confirmation of this J-shaped relationship is required with objective measures of energy intake in studies designed to assess intake across well-defined physical activity levels.

Of interest to this review are the studies that used preload challenges or macronutrient manipulations to examine whether differences exist in the ability to adjust energy intake after previous food intake or in meals that vary in composition. Three studies demonstrated that physically active individuals have a better ability to make adjustments in energy intake following a high-energy preload [19, 46, 48], suggesting increased sensitivity to previous energy intake (e.g. greater satiety). Another preload study also found more accurate energy compensation in active individuals, where the no-preload condition led to an increase in energy intake in active individuals but not in inactive individuals [37]. In line with these studies, one study found that exercise training led to a reduction in meal size at a high-energy density meal but not at a low-energy density meal [40]. This also supports the proposition of increased sensitivity to the energy density of foods, but this time during a meal (e.g. greater satiation). Interestingly, in this study it appeared that women may have been more susceptible to the effect than men. Therefore, further studies in males and females are required to confirm this finding and the potential interaction between physical activity and energy density on the sensitivity of appetite control. Nonetheless, these data support a J-shaped relationship between physical activity level and energy intake, and suggest a better ability to regulate energy intake with increasing levels of physical activity.

Despite the effects observed following a preload, there was no consistent effect of physical activity level on energy compensation immediately after an exercise bout or over several hours or days after exercise [27, 33, 35, 36, 38]. These results do not support a recent meta-analysis that found that absolute energy intake after acute exercise was greater in active individuals than in those who were less active [21]. However, this analysis reported only absolute energy intake and not energy compensation. In fact, Charlot and Chapelot [27] report in their study on lean/fit and fat/unfit men that energy compensation after exercise was highly variable, and they found no clear differences between groups. This raises the concern of the reliability of the measure of energy compensation (discussed in Sect. 4.3). Nevertheless, in the short-term, it appears that in physically active individuals, the regulation of energy intake may be more sensitive to previous food intake than to exercise.

### Differences in the Proposed Mechanisms of Appetite Control

Eating behaviour is influenced by several proposed mechanisms, one of which is appetite-related peptides. Acute exercise and exercise training also affect these peptides [53, 54]. The studies that measured the peptide response to food intake found lower postprandial insulin levels [34, 43, 47, 49] and higher postprandial GLP-1 levels [34] (and tendency [47]) in active individuals. An emphasis on insulin will be given, as it was the most commonly measured hormone in the studies within the review. Interestingly, the same subjects who showed a preload effect in the study by Martins et al. [48] also showed an improvement in insulin sensitivity [47]. Additionally, the aerobic training group in the study by Guelfi et al. [43] had significantly lowered postprandial insulin and improved insulin sensitivity, with concomitant changes in postprandial fullness. However, the resistance-training group in the same study had a tendency for lower postprandial insulin (*P* = 0.066) and also had improved insulin sensitivity after training, without an effect on postprandial appetite ratings, while another study that showed a preload effect after 6 weeks of training did not find a significant improvement in insulin sensitivity [46]. Despite the relationship between insulin and appetite control not being consistent in the above studies, a meta-analysis by Flint et al. [55] proposed that insulin resistance could lead to disrupted satiety signalling. This meta-analysis showed that postprandial insulin was associated with satiety in individuals with a healthy weight but not in overweight individuals; however, it did not take into account the physical activity status of the participants, nor their body composition (fat mass and fat-free mass).

Measuring body composition, rather than just BMI, has become important in understanding the mechanisms affecting eating behaviour, as fat-free mass (but not fat mass) was found to be associated with daily energy intake and meal size in overweight and obese individuals [56]. In addition to appetite signals from adipose tissue and gut hormones, Blundell et al. [56] proposed a role for fat-free mass and resting metabolic rate as drivers of food intake. Differences in body composition were apparent in the cross-sectional studies, as six reported lower body fat percentage in active individuals [25, 33–37], despite only two reporting a lower BMI [25, 35]. Three of the former studies reported enhanced appetite control in terms of more accurate energy compensation [33, 35, 37]. No cross-sectional studies compared lean and overweight active individuals, thus a question arises as whether ‘fat but fit’ individuals would have enhanced appetite control. Four training studies conducted in overweight participants reported improvements in appetite control post-intervention (but also showed significant reductions in fat mass) [40, 41, 43, 48]. Overall, these studies indicate that differences in body composition and insulin sensitivity may be factors promoting more sensitive appetite control in active individuals. Furthermore, a recent study found faster gastric emptying in active males than in inactive males [57], proposing another mechanism by which appetite control (i.e. satiety signalling) could be better regulated in physically active individuals. More studies are required to elucidate the mechanisms involved in the appetite control differences between active and inactive individuals, such as body composition, postprandial satiety and hunger peptides, insulin (and possibly leptin [9, 10]) sensitivity and gastric emptying, in addition to resting metabolic rate [40, 56] and substrate oxidation [58], which were not covered in this review.

### Methodological Considerations

A number of points regarding the methodologies used in the studies included in this review need addressing. In the cross-sectional studies, the definitions used for active and inactive individuals varied markedly. For example, some studies used only a self-rated measure (‘yes or no’ question [29] or a Likert scale [28, 30, 32]) or a self-reported measure (physical activity questionnaires [26, 37] or diaries/recalls [19, 33]) instead of objectively assessing physical activity via accelerometry. This may have confounded the results of the active groups from participants overestimating their physical activity habits [59, 60]. Moreover, some studies only used VO_2max_ [27, 34] to define the active groups, which may not have reflected all aspects of physical activity (e.g. low- to moderate-intensity activity) [61]. Clear definitions of activity levels should be set in place to allow future studies to investigate appetite and energy intake across these defined levels. Along these lines, the studies in this review preclude us from distinguishing the effects of the several aspects of physical activity—such as time spent in low, moderate and vigorous activities, cardiovascular fitness and activity-related energy expenditure—on appetite control. In addition, future studies should assess all components of energy intake and energy expenditure in order to determine their influence on eating behaviour, particularly in light of recent evidence suggesting a plateau in daily energy expenditure above a certain threshold of physical activity [62]. This would allow us to tease out whether changes in cardiovascular fitness and/or physical activity energy expenditure are most important for appetite control. Secondly, food intake was assessed both in laboratory conditions (using test meals) and in free-living conditions (using an FFQ or food diaries). Test meals are known to be a rigorous method of assessing energy intake (under controlled laboratory conditions), but food diaries—despite providing a longer window of observation of ‘real world’ feeding patterns—may lead to underreporting and biased results [59]. It should be noted that the short-term results (daily energy intake) observed in the preload studies were based on food diaries [19, 37, 46, 48]. These data should be replicated in more rigorous conditions to confirm the observed effects. Thirdly, the within-subject consistency (i.e. test–retest reliability) and between-subject consistency (i.e. interindividual variability) in energy compensation following preload intake is often not acknowledged in studies, and this should be addressed in light of recent studies demonstrating marked interindividual variability [27, 63–65] and modest test–retest reliability [66] in energy compensation following acute exercise. The composition of the preloads and tests meals should also be further examined to determine whether physical activity enhances the sensitivity to energy density or to specific macronutrients. Finally, the sample size in most of the studies was small, which may have resulted in non-significant results and caused relatively small but important effects to be overlooked. The studies were also not designed to test the effects of sex, body composition (lean versus overweight) and exercise mode; therefore, this does not allow us to determine specific criteria or characteristics eliciting the reported effects (or lack thereof).

### Review Limitations

This review included a limited number of studies assessing a broad range of appetite-related measures between active and inactive individuals, using various definitions. This may have led to some of the inconsistent patterns or lack of effects observed. Physical activity encompasses not only exercise training but also activities of daily living, and, as most definitions were based on a minimal level of moderate-intensity structured exercise, the studies included in this review leaned towards a comparison between exercise-trained and untrained individuals. Therefore, these results should be interpreted with caution while more studies assessing all facets of habitual physical activity become available. Clearly, there is a lot more work to be done to elucidate the effects of physical activity and exercise on the appetite control system.

## Conclusion

It can be concluded from this review that habitually active individuals appear to have increased sensitivity to the energy density of foods, in comparison with inactive individuals, despite the lack of observable group differences in subjective appetite ratings. This review also supports the formulation that the relationship between physical activity level and energy intake may be non-linear, as reflected by the J-shaped curve obtained from analysis of standardized energy intake scores. The mechanisms underlying this effect are not known but could include differences in body composition (fat mass and fat-free mass), postprandial hunger or satiety peptides, or sensitivity to tonic peptides, such as insulin or leptin. This characteristic of active individuals could mitigate the risk of overconsumption in an energy-dense food environment. Further studies are required to confirm these findings.

## Electronic supplementary material

Below is the link to the electronic supplementary material.

Supplementary material 1 (DOCX 30 kb)

Supplementary material 2 (DOCX 22 kb)

Supplementary material 3 (DOCX 64 kb)
