# Supplementary material for: Does Habitual Physical Activity Increase the Sensitivity of the Appetite Control System? A Systematic Review
Source: Sports Med. 2016 Mar 22;46(12):1897–919. doi: 10.1007/s40279-016-0518-9 (PMC5097075; doi:10.1007/s40279-016-0518-9)
Supplement: Supplementary file 2 — Supplementary material 2 (DOCX 22 kb) [file 40279_2016_518_MOESM2_ESM.docx]

Does habitual physical activity increase the sensitivity of the appetite control system? A systematic review, Sports Medicine

Kristine Beaulieu^*^, Mark Hopkins, John Blundell & Graham Finlayson

*Corresponding author. School of Psychology, University of Leeds; email: k.beaulieu14@leeds.ac.uk

**Electronic Supplementary Material Appendix S2.** Detailed search strategy

OvidSP Medline

1. Motor activity/
2. Exercise/
3. Oxygen consumption/
4. Physical Fitness/
5. Exercise tolerance/
6. Exercise test
7. Physical endurance
8. Physical activity
9. Physical performance
10. Aerobic
11. Aerobic capacity
12. Training
13. Maximal VO2
14. Physical capacity
15. or/1-14
16. Appetite/
17. Feeding behavior/ or food preferences/
18. Hunger
19. Satiety
20. Satiation
21. Fullness
22. Motivation to eat
23. Food choice
24. Food selection
25. Desire to eat
26. Palatability
27. Food reward
28. Hedonic
29. Liking
30. Wanting
31. or/1-15
32. Energy intake/
33. Diet/
34. Calori* intake
35. Food intake
36. Meal size
37. Energy compensation
38. Energy density
39. Dietary proteins/ or dietary fats/ or dietary carbohydrates/
40. Macronutrient
41. or/1-9
42. Gut hormone*
43. Gut peptide*
44. Peptide YY or PYY
45. Ghrelin
46. Glucagon-like peptide-1 or GLP-1
47. Pancreatic polypeptide or PP
48. Leptin
49. Insulin
50. Cholecystokinin or CCK
51. Or/ 1-9
52. 41 OR 51
53. 31 AND 52
54. 15 AND 53
55. Limit 54 to (English language and humans and 19-64 years)
