# Supplementary material for: Does Habitual Physical Activity Increase the Sensitivity of the Appetite Control System? A Systematic Review
Source: Sports Med. 2016 Mar 22;46(12):1897–919. doi: 10.1007/s40279-016-0518-9 (PMC5097075; doi:10.1007/s40279-016-0518-9)
Supplement: Supplementary file 3 — Supplementary material 3 (DOCX 64 kb) [file 40279_2016_518_MOESM3_ESM.docx]

Does habitual physical activity increase the sensitivity of the appetite control system? A systematic review, Sports Medicine

Kristine Beaulieu^*^, Mark Hopkins, John Blundell & Graham Finlayson

*Corresponding author. School of Psychology, University of Leeds; email: k.beaulieu14@leeds.ac.uk

**Electronic Supplementary Material Table S1.** Risk of bias assessment

|  | **Sequence generation** | **Allocation concealment** | **Blinding participants and personnel** | **Blinding outcome assessors** | **Incomplete outcome data** | **Selective outcome** | **Other source of bias** |
| --- | --- | --- | --- | --- | --- | --- | --- |
| **Cross-sectional studies** | | | | | | | |
| Apolzan et al. (2009) [[23](#_ENREF_23)] | N/A | N/A | Unclear risk | Unclear risk | Low risk | Low risk | High risk |
| Catenacci et al. (2014) [[24](#_ENREF_24)] | N/A | N/A | Unclear risk | Unclear risk | Low risk | Low risk | High risk |
| Charlot & Chapelot (2013) [[31](#_ENREF_31)] | Unclear risk | N/A | High risk | Unclear risk | Low risk | Low risk | High risk |
| Deshmukh-Taskar et al. (2007) [[25](#_ENREF_25)] | N/A | N/A | Unclear risk | Unclear risk | High risk | Low risk | High risk |
| Georgiou et al. (1996) [[26](#_ENREF_26)] | N/A | N/A | Unclear risk | Unclear risk | Unclear risk | Low risk | High risk |
| Gregersen et al. (2011) [[27](#_ENREF_27)] | N/A | N/A | Unclear risk | Unclear risk | Low risk | Low risk | High risk |
| Harrington et al. (2013) [[28](#_ENREF_28)] | N/A | N/A | Unclear risk | Unclear risk | Low risk | Low risk | High risk |
| Jago et al. (2005) [[29](#_ENREF_29)] | N/A | N/A | Unclear risk | Unclear risk | High risk | Low risk | High risk |
| Jokisch et al. (2012) [[32](#_ENREF_32)] | Unclear risk | N/A | High risk | Unclear risk | Low risk | Low risk | High risk |
| Long et al. (2002) [[33](#_ENREF_33)] | Unclear risk | N/A | Low risk | Unclear risk | Low risk | Low risk | High risk |
| Lund et al. (2013) [[34](#_ENREF_34)] | N/A | N/A | Unclear risk | Unclear risk | Low risk | Low risk | High risk |
| Rocha et al. (2013) [[35](#_ENREF_35)] | Unclear risk | N/A | High risk | Unclear risk | Low risk | Low risk | High risk |
| Rocha et al. (2015) [[36](#_ENREF_36)] | Unclear risk | N/A | High risk | Unclear risk | Low risk | Low risk | High risk |
| Van Walleghen et al. (2007) [[30](#_ENREF_30)] | Unclear risk | N/A | High risk | Unclear risk | Low risk | Low risk | High risk |
| **Exercise-training studies** | | | | | | | |
| Alkahtani et al. (2014) [[49](#_ENREF_49)] | Unclear risk | N/A | High risk | Unclear risk | Low risk | Low risk | High risk |
| Bryant et al. (2012) [[37](#_ENREF_37)] | N/A | N/A | Unclear risk | Unclear risk | Unclear risk | Low risk | High risk |
| Caudwell et al. (2013) [[38](#_ENREF_38)] | N/A | N/A | Unclear risk | Unclear risk | Unclear risk | Low risk | High risk |
| Caudwell et al. (2013) [[39](#_ENREF_39)] | N/A | N/A | Unclear risk | Unclear risk | Unclear risk | Low risk | High risk |
| Cornier et al. (2012) [[40](#_ENREF_40)] | N/A | N/A | Unclear risk | Unclear risk | Low risk | Low risk | High risk |
| Guelfi et al. (2013) [[41](#_ENREF_41)] | Unclear risk | Unclear risk | High risk | Unclear risk | Low risk | Low risk | High risk |
| Jakicic et al. (2011) [[50](#_ENREF_50)] | Unclear risk | Unclear risk | High risk | Unclear risk | Low risk | Low risk | High risk |
| King et al. (2008) [[43](#_ENREF_43)] | N/A | N/A | Unclear risk | Unclear risk | Low risk | Low risk | High risk |
| King et al. (2009) [[42](#_ENREF_42)] | N/A | N/A | Unclear risk | Unclear risk | Unclear risk | Low risk | High risk |
| Martins et al. (2007) [[65](#_ENREF_65)] | N/A | N/A | Low risk | Unclear risk | Low risk | Low risk | High risk |
| Martins et al. (2010) [[44](#_ENREF_44)] | N/A | N/A | Unclear risk | Unclear risk | Low risk | Low risk | High risk |
| Martins et al. (2013) [[45](#_ENREF_45)] | N/A | N/A | Unclear risk | Unclear risk | Low risk | Low risk | High risk |
| Rosenkilde et al. (2013) [[47](#_ENREF_47)] | Low risk | N/A | Unclear risk | Unclear risk | Low risk | Low risk | High risk |
| Shaw et al. (2010) [[48](#_ENREF_48)] | Low risk | N/A | High risk | Unclear risk | Low risk | Low risk | High risk |
